# Ferroelectricity in Atomic Layer Deposited Wurtzite Zinc Magnesium Oxide Zn_1–*x*_Mg_*x*_O

**DOI:** 10.1021/acs.nanolett.5c02005

**Published:** 2025-06-09

**Authors:** Benjamin L. Aronson, Kyle P. Kelley, Ece Gunay, Ian Mercer, Bogdan Dryzhakov, Jon-Paul Maria, Elizabeth C. Dickey, Susan Trolier-McKinstry, Jon F. Ihlefeld

**Affiliations:** † Department of Materials Science and Engineering, 189555 University of Virginia, Charlottesville, Virginia 22904, United States; ‡ Center for Nanophase Materials Sciences, 6146 Oak Ridge National Laboratory, Oak Ridge, Tennessee 37381, United States; § Department of Materials Science and Engineering, 6612 Carnegie Mellon University, Pittsburgh, Pennsylvania 15213, United States; ∥ Department of Materials Science and Engineering, The Pennsylvania State University, University Park, Pennsylvania 16802, United States; ⊥ Charles L. Brown Department of Electrical and Computer Engineering, University of Virginia, Charlottesville, Virginia 22904, United States

**Keywords:** Ferroelectric, Thin Films, Wurtzite, Atomic Layer Deposition, Zinc Magnesium Oxide

## Abstract

Conformal deposition of wurtzite ferroelectrics, which is needed for their use in scaled nonvolatile memories, is challenging using current physical vapor deposition techniques. To overcome the conformality barrier, this work demonstrates ferroelectricity in wurtzite Zn_1–*x*_Mg_*x*_O thin films prepared by plasma-enhanced atomic layer deposition, which is a non-line-of-sight deposition method. Films ranging in composition from *x* = 0.00 to *x* = 0.58 are predominantly wurtzite phase and exhibit a (0001)-texture. Increasing the magnesium content decreases the *c*/*a* ratio, increases the optical bandgap energy, increases the piezoelectric response, and enables polarization reversal. Clear polarization switching is demonstrated in 50 nm thick Zn_1–*x*_Mg_*x*_O films by piezoresponse force microscopy in compositions containing *x* = 0.46 and *x* = 0.58.

## Graphical abstract

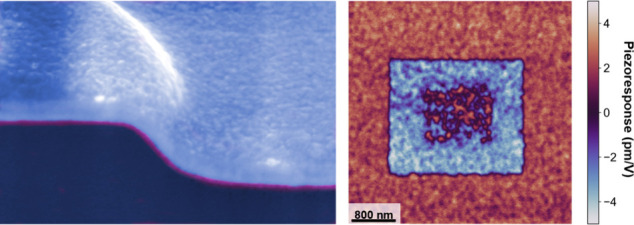

Widespread adoption of ferroelectric nonvolatile memory into scaled microelectronics holds promise for improved power efficiency, decreased read/write times, and lower cost per memory bit. Currently, however, perovskite ferroelectrics such as lead zirconate titanate (PbZr_1–*x*_Ti_*x*_O_3_) and barium titanate (BaTiO_3_) are hindered by inadequate scaling and challenging complementary metal-oxide-semiconductor (CMOS) back-end-of-line (BEOL) integration. Ideally, ferroelectrics can be processed at temperatures below 400 °C, have chemical compatibility with semiconductors like silicon, and be conformally grown on complex geometries. Hafnia-based ferroelectrics, first reported in 2011,^1^ offer advantages in thickness scaling, low-process temperatures, chemical compatibility, and conformal growth relative to the perovskites. However, polymorphism, ferroelastic switching, and high concentrations of point defects result in performance instabilities.^2–6^ In contrast, ferroelectric wurtzites have a single polymorph without ferroelastic domains, while maintaining BEOL-compatible processing temperatures.^7–9^ Moreover, ferroelectric wurtzites have large remanent polarizations on the order of 75–150 μC/cm^2^,^7–12^ maintain polarization reversal in sub-10 nm film thicknesses,^13,14^ and show long retention lifetimes.^15,16^ Altogether, these properties make wurtzites an appealing candidate for integrating ferroelectric nonvolatile memory devices, such as ferroelectric random access memory (FeRAM), ferroelectric tunnel junctions (FTJs), and ferroelectric diodes, into mainstream semiconductor technologies.^17^

Crystals adopting the wurtzite structure (space group *P*6_3_ *mc*) have long been viewed as polar but not ferroelectric due to an inability to reverse the polarization without undergoing dielectric breakdown. This perspective changed in 2019 when ferroelectric switching of wurtzite aluminum scandium nitride, Al_1–*x*_Sc_*x*_N, thin films was demonstrated.^7^ Thereafter, ferroelectricity has been observed in additional AlN-based compositions,^8,11,12,18,19^ alloyed GaN,^10^ and alloyed ZnO,^9,20^ whereby cation substitution is speculated to generate local strain^12,21^ and bond ionicity fluctuations.^22^ This disorder modifies the energy landscape, reducing the coercive field and allowing polarization reversal along the polar *c*-axis of the wurtzite structure. Mg-substituted ZnO (Zn_1–*x*_Mg_*x*_O), in particular, has a low coercive field (2–4 MV/cm) relative to most other wurtzite ferroelectrics, demonstrated integrability on flexible polymer substrates, and deposition temperatures as low as room temperature.^7–10,12,20,23^

The majority of ferroelectric wurtzite thin films, including Zn_1–*x*_Mg_*x*_O, have been fabricated using physical vapor deposition (PVD) techniques, including sputtering^7–9,12,20^ and molecular-beam epitaxy (MBE).^10,11,24,25^ These growth methods are largely line-of-sight with limited conformality. Due to the demand for high-aspect-ratio structures, such as deep trench capacitors, in high density ferroelectric nonvolatile memory devices, there is a need to conformally deposit ferroelectric wurtzites.^17,26,27^ Atomic layer deposition (ALD) utilizes self-limiting reactions of organometallic precursors and coreactants in sequential cycles of reactant doses and purges. Due to the high growth pressure and self-limiting chemical vapor nature of ALD, conformal and uniform films with precise thickness control can be achieved. This offers an advantage in coating complex surfaces, such as high-aspect-ratio structures, relative to PVD processes. Therefore, ALD presents an opportunity for conformally depositing ferroelectric wurtzites into 3D geometries. While ferroelectricity in ALD-grown wurtzites has not been reported thus far, ALD growth of Zn_1–*x*_Mg_*x*_O with *x* values large enough to observe ferroelectricity has been investigated for use as a buffer layer in photovoltaics.^28–30^ Thus, the prior work illustrates promise in investigating this material as an ALD-grown wurtzite ferroelectric.

In this Letter, ferroelectricity is demonstrated in nominally 50 nm thick ALD-grown Zn_1–*x*_Mg_*x*_O thin films. Zn_1–*x*_Mg_*x*_O of varying composition between *x* = 0 and *x* = 0.58 were deposited onto platinized silicon substrates (50 nm (111)-oriented Pt/5 nm Ti/SiO_2_/(001)-oriented Si) using a plasma-enhanced ALD process. Magnesium oxide was introduced into the ZnO through the use of supercycles of the ALD precursors. For example, an *x* = 0.30 composition was achieved with a dose ratio of 1:3 MgO:ZnO repeated for 80 total supercycles, resulting in a 50 nm thick film. The magnesium content obtained for each MgO:ZnO supercycle ratio used in the Zn_1–*x*_Mg_*x*_O film growth is shown in Figure S1; the incorporated Mg scales with a nearly linear monotonic trend in terms of the supercycle ratio. Crystalline phase, texture, lattice parameters, and mosaicity were assessed by X-ray diffraction (XRD), revealing a structural dependence upon magnesium substitution. Film thickness and optical bandgap energies were determined using variable angle spectroscopic ellipsometry (VASE), with additional morphological information provided by scanning electron microscopy (SEM) and atomic force microscopy (AFM), and structural information and chemistry provided by scanning/transmission electron microscopy (S/TEM) and energy dispersive spectroscopy (EDS). Rutherford backscattering spectrometry (RBS) was used to measure the magnesium content (*x*) in the films. To assess the ferroelectric behavior, piezoresponse force microscopy (PFM) was performed, in which polarization switching was demonstrated in the two highest magnesium-containing compositions, *x* = 0.46 and 0.58.

Figure 1(a) shows the symmetric out-of-plane 2θ-ω XRD patterns measured for a composition series of 50 nm thick Zn_1–*x*_Mg_*x*_O films deposited on (111) Pt/Ti/SiO_2_/Si. The only diffraction peaks present between 32° and 44° in 2θ correspond to the wurtzite Zn_1–*x*_Mg_*x*_O 0002 and Pt 111 reflections. All films deposited with compositions between *x* = 0 (0:1 MgO:ZnO) and *x* = 0.58 (1:1 MgO:ZnO) have reflections that may only be attributed to the wurtzite structure and the substrate. At a cycle ratio of 2:1 MgO:ZnO, the rocksalt Zn_1–*x*_Mg_*x*_O 111 reflection is observed, as shown in Figure S2 in the Supporting Information. The presence of the wurtzite Zn_1–*x*_Mg_*x*_O 0002 reflection with no other wurtzite reflections for magnesium concentrations at and below *x* = 0.58 is indicative of preferred *c*-axis texturing, which is necessary for out-of-plane ferroelectric switching as the spontaneous polarization in wurtzites exists only along the polar *c*-axis. 2*θ*_*χ*_ XRD measurements are utilized to assess the in-plane lattice spacings and validate that the deposited films were *c*-axis textured (Figure 1(b)). A strong wurtzite 1010 reflection is present with no additional wurtzite reflections. Given that the (0002) and (1010) crystallographic planes are perpendicular to one another, this further confirms that the Zn_1–*x*_Mg_*x*_O films are primarily composed of a singular out-of-plane *c*-axis textured wurtzite structure. No rocksalt phase reflections are observed in either the out-of-plane or in-plane XRD measurements, suggesting that films containing up to 58% magnesium can be stabilized primarily into the wurtzite phase when prepared by ALD.

**Figure 1:**
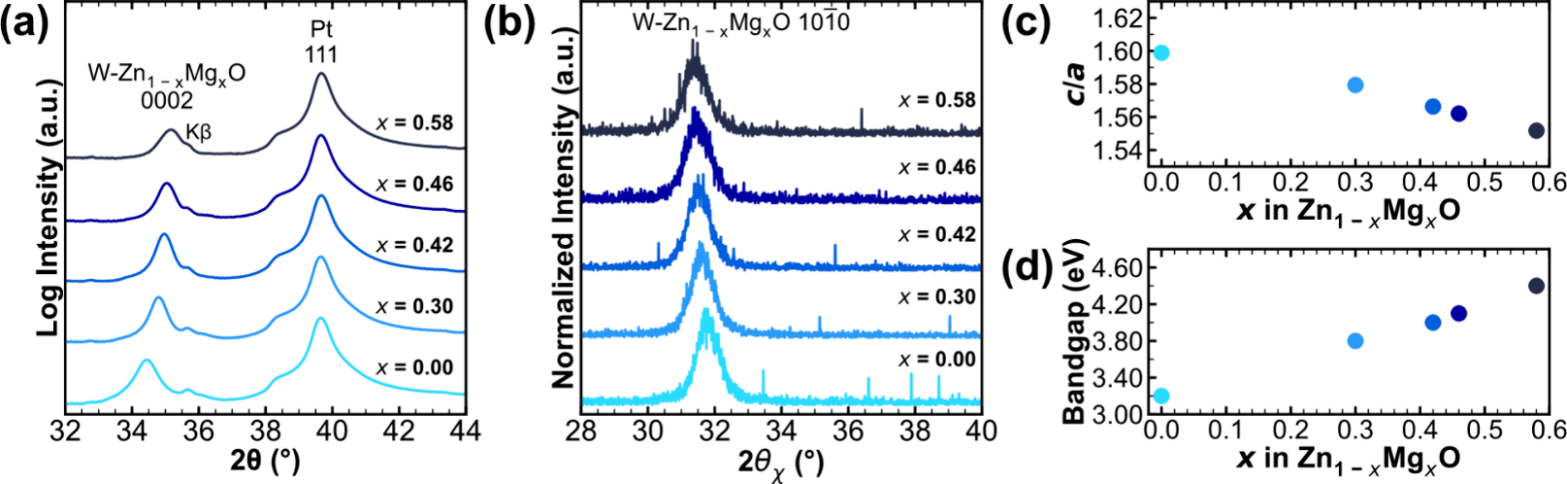
(a) Symmetric out-of-plane 2θ–ω X-ray diffraction patterns, (b) in-plane 2θ_χ_ X-ray diffraction patterns, (c) *c*/*a* lattice parameter ratio, and (d) optical bandgap energies across the Zn_1–*x*_Mg_*x*_O composition series for *x* = 0, 0.30, 0.42, 0.46, and 0.58.

The impact of magnesium substitution on the lattice parameters of the Zn_1–*x*_Mg_*x*_O films was investigated, as structural disorder and distortions are factors suggested to enable switching.^21,31^ With increasing magnesium content, the out-of-plane 0002 reflection shifts to higher 2θ angles and the in-plane 1010 reflection to lower 2*θ*_*χ*_ angles. Consequently, increasing the magnesium content in the ALD Zn_1–*x*_Mg_*x*_O leads to a monotonic decrease in the *c*- and an increase in the *a*-lattice parameters (Figure S3). The addition of MgO to ZnO thus drives a simultaneous in-plane expansion and out-of-plane contraction in the wurtzite unit cell and is consistent with observations made on Zn_1–*x*_Mg_*x*_O films prepared by multiple growth techniques.^9,32–34^ The *c*/*a* ratio is inversely correlated to the internal parameter *u*, which is indicative of the wurtzite tetrahedra distortion along [0001]. Figure 1(c) shows that the *c*/*a* value undergoes a continuous decrease from 1.60 to 1.55 with increasing magnesium concentration. A decreased *c*/*a* value, and corresponding increase in *u*, has been shown in previous studies to correlate with ferroelectric switching.^7,9^ This was postulated to be a consequence of the structural similarity to the nonpolar, hexagonal boron nitride phase (space group *P*6_3_/*mmc*), which was proposed to act as a transition state during polarization reversal.^7,9,31^ However, other polarization reversal mechanisms that do not require transiting through the hexagonal boron nitride phase have since been reported.^12,21,35,36^

The structure was further assessed using XRD ω rocking curve scans of the 0002 wurtzite reflection. The full width at half-maximum (FWHM) of the ω rocking curves across the composition range are shown in Figure S4. Compositions from *x* = 0 to *x* = 0.42 have a FWHM of 3.5°, which is identical to that of the (111)-textured platinum substrate. For *x* = 0.46 and *x* = 0.58, a FWHM increase up to 4.4° is observed, revealing that mosaicity increases with greater magnesium concentrations for these growth conditions. The greater structural distortion observed in films of *x* = 0.46 and *x* = 0.58 may promote ferroelectric switching, as structural distortions can enable polarization reversal in Zn_1–*x*_Mg_*x*_O.

In addition to generating structural distortions and chemical disorder in the film, magnesium substitution increases the bandgap in the ZnO-MgO system as the bandgap of MgO (∼7.8 eV)^37^ is greater than that of ZnO (∼3.3 eV).^38^ An increased bandgap may thus allow for Zn_1–*x*_Mg_*x*_O films to withstand the application of larger electric fields. The wavelength-dependent extinction coefficient (*k*), as determined by VASE, was used to calculate the absorption coefficient (α) for each film composition. Direct optical bandgap energies were determined using Tauc fitting, in which (*αhυ*)^2^ is plotted as a function of incident photon energy (Figure S5) with the absorption edge extrapolated to zero. Figure 1(d) shows that the bandgap energies increase monotonically from 3.2 eV at *x* = 0 up to 4.4 eV at *x* = 0.58. This trend aligns with observations in prior studies showing that magnesium substitution in ZnO increases the bandgap,^9,28,32,33,39^ and demonstrates that higher bandgaps than previously reported^9,30,33^ may be obtained in films prepared by ALD where large magnesium concentrations can be incorporated without significant phase segregation.

Local structural characterization was conducted to provide a greater understanding of the film microstructure. Figure 2(a) presents a cross-sectional bright-field TEM image of the highest MgO content film that is phase-pure by XRD, Zn_0.42_Mg_0.58_O. A partially columnar microstructure is observed as not all grains extend continuously through the entire film thickness. The selected area electron diffraction (SAED) pattern inset in Figure 2(a) shows the primary diffraction spots corresponding to the characteristic reflections of the wurtzite structure. Additionally, the out-of-plane 0002 reflection confirms *c*-axis oriented growth, while the presence of both 1100 and 1120 type in-plane reflections indicate a fiber textured film, which is expected for templating from the fiber-textured electrode layer. Figure 2(b) displays STEM-EDS elemental maps, showing a uniform elemental distribution within the bulk of the film, but magnesium enrichment near the interface with the platinum electrode that is approximately 4.5 nm thick. This local compositional variation may be due to either diffusion during the growth or a nucleation barrier for ZnO on the platinum surface, but remains uncertain. The HRTEM and corresponding FFTs of this interface are displayed in Figure S6. This magnesium enrichment corresponds with locally cubic (rocksalt) regions in this highest Mg-content film and there is an orientation relationship between the rocksalt (111) and wurtzite (0001) planes. This illustrates that while the XRD did not reveal secondary phases, there are small secondary phases present in the film with this high magnesium content. Figure 2(c) provides atomic-scale resolution high angle annular dark field (HAADF) images from the bulk of the film acquired using STEM mode. Local differentiated differential phase contrast (dDPC) images combined with the HAADF signal (presented to the right) provide information about both the anion and cation sublattices and reveal the presence of both oxygen-polar orientations (polarization vector pointing toward the Zn_1–*x*_Mg_*x*_O/Pt interface) and metal-polar orientations (polarization up) in the as-deposited state.

**Figure 2:**
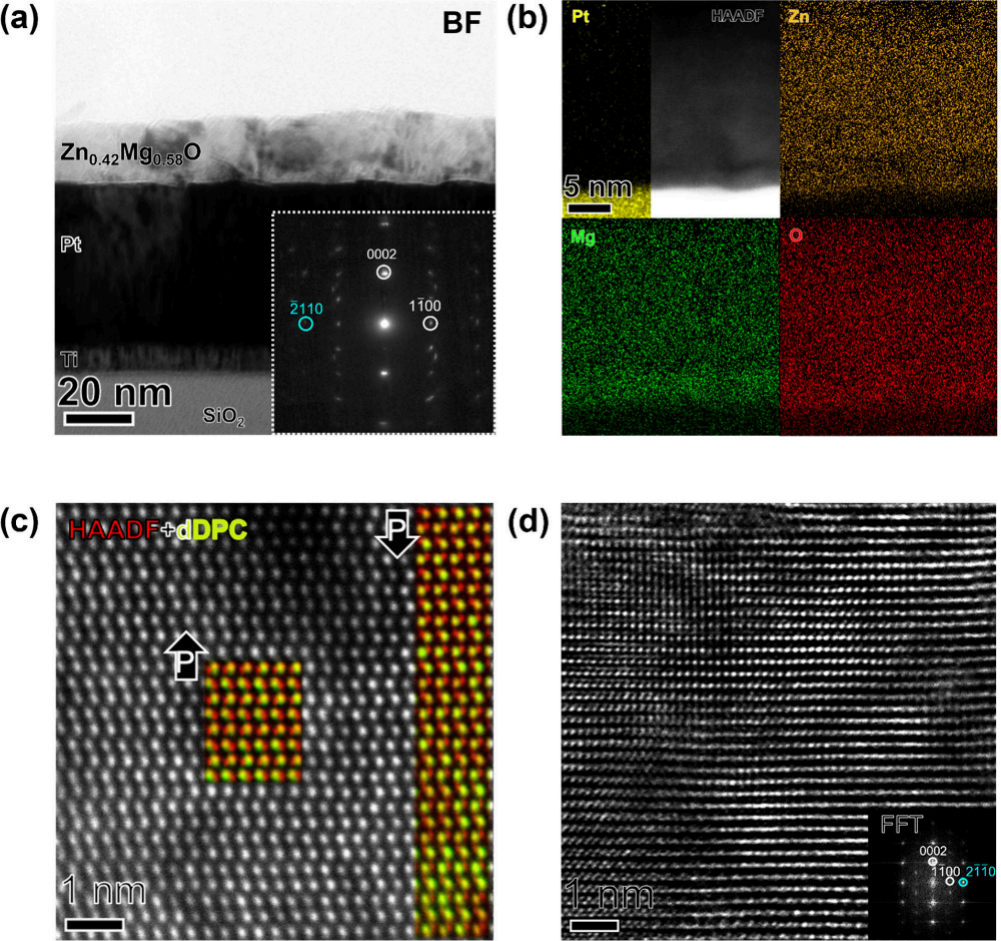
(a) Bright-field TEM image of the Zn_0.42_Mg_0.58_O/Pt/Ti/SiO_2_/Si stack with selected area diffraction pattern of the film (inset). (b) STEM-EDS elemental map indicating zinc and magnesium distribution throughout the thickness of the film, (c) STEM-HAADF atomic resolution image of the film structure, overlaid with dDPC showing the polar alignment of oxygen anions (red) and cations (green). (d) High-resolution transmission electron microscopy (HRTEM) image of the Zn_0.42_Mg_0.58_O film in the bulk region. The inset displays the corresponding fast Fourier transform (FFT).

High-resolution TEM (HRTEM) shown in Figure 2(d) reveals evidence of crystalline mosaicity in the structure as the atomic planes are not perfectly flat across the imaged region. The structural distortions present in the image are consistent with the large FWHM in ω from the XRD measurements shown in this Figure S4. Moreover, the fast Fourier transform (FFT) inset is consistent with the wurtzite crystal structure. Ultimately, the TEM-based microstructural analyses conducted on the Zn_0.42_Mg_0.58_O film indicate a highly textured, predominantly wurtzite mixed-polarity film with small regions of rocksalt. This demonstrates that the *x* = 0.58 composition is near the solubility limit of the metastable wurtzite region at these growth conditions.

TEM analysis was also carried out on the *x* = 0.46 composition and comparisons may be made between these two films. Consistent with the *x* = 0.58 composition, the *x* = 0.46 composition exhibited a predominantly out-of-plane *c*-textured wurtzite structure with magnesium enrichment and rocksalt phase evolution along the Zn_0.54_Mg_0.46_O/Pt interface (Figure S7­(a–c)). Evidence of crystalline mosaicity was similarly present in the *x* = 0.46 composition, as displayed in the HRTEM image in Figure S8. However, in contrast to the *x* = 0.58 composition, the *x* = 0.46 composition exhibited an oxygen-polar columnar microstructure with grains extending through the full thickness of the film (Figure S9­(a,b)).

Ferroelectric and piezoelectric properties were examined via piezoresponse force microscopy integrated with quadrature phase differential interferometry.^40^ This interferometric-based sensing enables quantitative measurements of AFM tip displacements measured in PFM, reducing artifacts such as electrostatic forces between the cantilever and sample. Figure 3(a–e) displays the piezoresponse across the Zn_1–*x*_Mg_*x*_O composition series from *x* = 0.00 to *x* = 0.58. The lowest average piezoresponse of 1.2 pm/V is observed for *x* = 0, featuring a predominantly oxygen-polar polarization state (polarization pointing into the page) with localized regions of reversed polarization. The oxygen-polar polarization orientation is maintained with increasing magnesium concentrations up to *x* = 0.46, with a corresponding monotonic increase in the piezoresponse up to 5.1 pm/V. For *x* = 0.58, the average piezoresponse decreases to 2.1 pm/V. This decrease in average piezoresponse coincides with a reemergence of local metal-polar domains (polarization pointing out of the page), which were observed in the STEM imaging. The increasing *d*_33_ trend with magnesium content observed here is consistent with that observed in pulsed laser deposited films,^34^ with the ALD-prepared films exhibiting slightly lower values for each composition. The reason for the difference is unclear but may be related to mixed polarity in the films, carbon retention, measurements taken on the bare film surface, and/or differences in composition.

**Figure 3:**
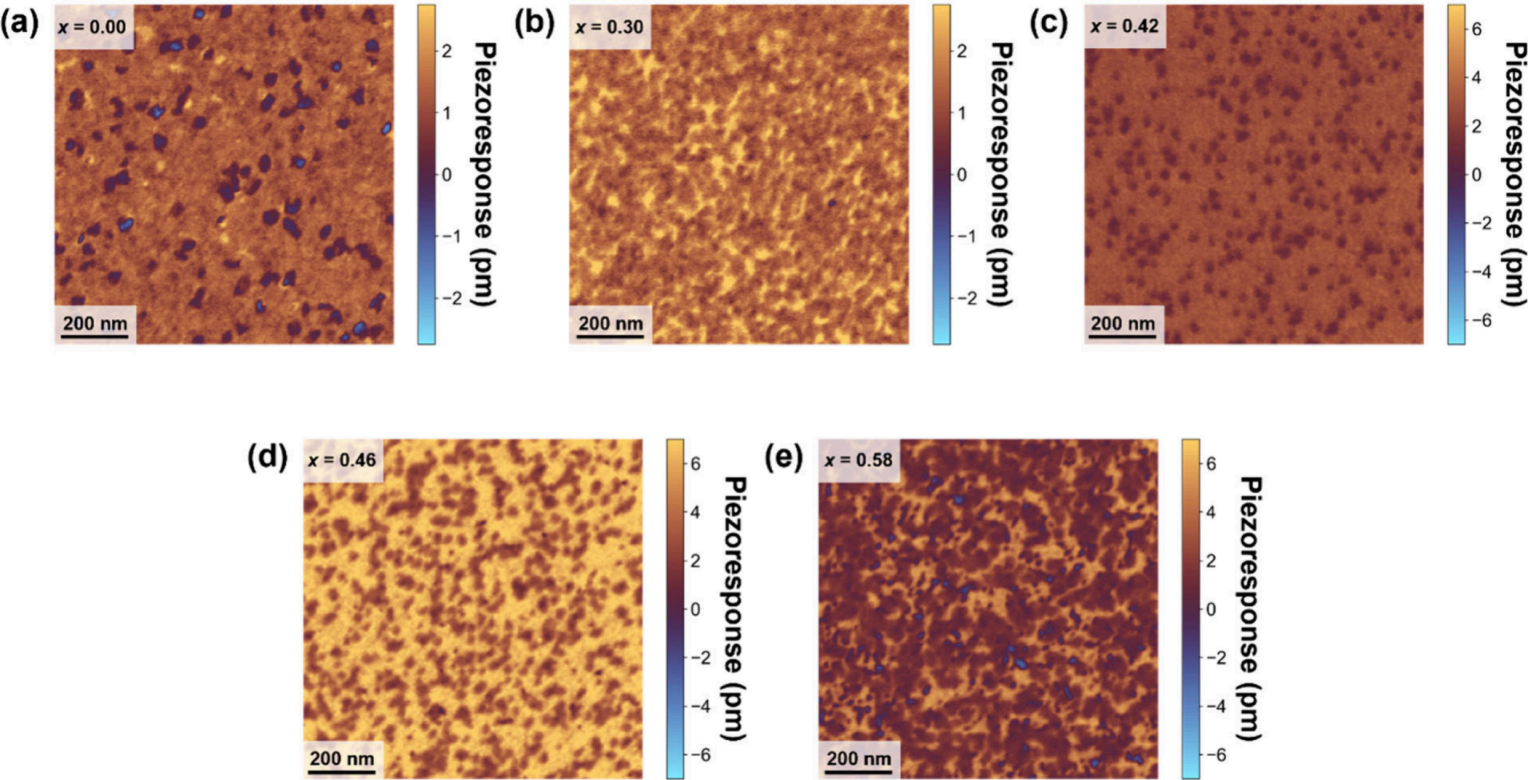
Piezoresponse force microscopy images for Zn_1–*x*_Mg_*x*_O films with magnesium concentrations of (a) *x* = 0.00, (b) *x* = 0.30, (c) *x* = 0.42, (d) *x* = 0.46, and (e) *x* = 0.58.

Ferroelectric polarization hysteresis measurements on macroscopic capacitors (50 μm diameter) were hindered by excessive leakage currents and dielectric breakdown below the coercive field. Therefore, to gain further insight into the local ferroelectric switching characteristics, PFM amplitude and phase hysteresis loops were measured. For *x* = 0.46 and *x* = 0.58, evidence of polarization reversal is indicated by 3.14 radian phase reversal and amplitude butterfly loops as shown in Figure 4(a,b). Ferroelectric switching has been reported with similar compositions in sputtered Zn_1–*x*_Mg_*x*_O thin films.^23^ The electrical conductivity was too high to obtain reliable measurements on the ALD-prepared Zn_1–*x*_Mg_*x*_O films with lower magnesium contents (i.e., *x* = 0, 0.30, and 0.42), as artifacts associated with Joule heating and large currents passing through the AFM tip interfered with the measurements. In contrast, ferroelectric switching has been demonstrated in sputtered Zn_1–*x*_Mg_*x*_O with magnesium concentrations as low as *x* = 0.08.^23^ This disparity between ALD and sputtered ferroelectric Zn_1–*x*_Mg_*x*_O is suspected to be due to fewer point defects in sputtered films and warrants further investigation.

**Figure 4:**
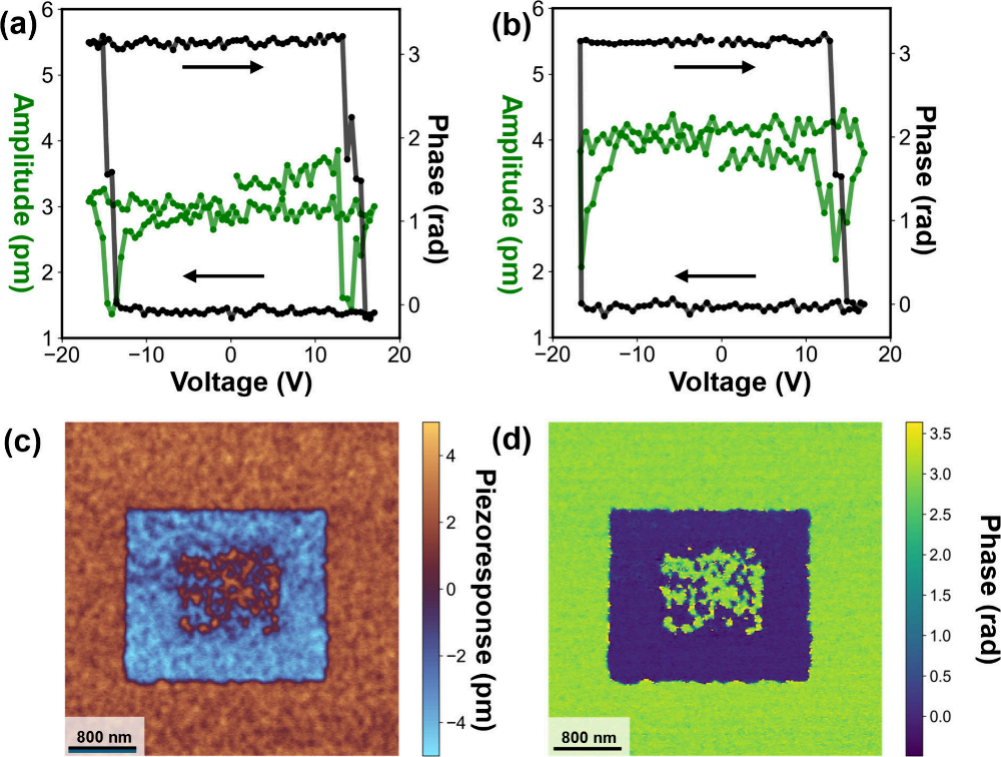
Switching spectroscopy displaying off-field amplitude (green) and phase (black) of locally switched Zn_1–*x*_Mg_*x*_O for (a) *x* = 0.46 and (b) *x* = 0.58. The (c) piezoresponse force microscopy and (d) phase images of ferroelectric domain lithography performed by applying a DC bias to the cantilever to locally switch polarization within specified regions for the *x* = 0.58 film. Bias voltages of −16 and +16 V were applied to the cantilever, resulting in polarization switching over areas of 2.5 and 1 μm, respectively.

In corroboration with the switching spectroscopy results, Figure 4(c,d) shows PFM and phase images, respectively, on the *x* = 0.58 composition film after the poling of 2.5 and 1.0 μm areas by applying −16 V and +16 V DC, respectively, to the AFM cantilever. Ferroelectric domain inversion is observed when applying a negative voltage to the cantilever. Switching the smaller region back to the original polarization state yields heterogeneous local domain switching. Homogeneous ferroelectric domain reversal is speculated to be prohibited by the applied voltage being very close to the coercive field, large pinning fields associated with pinning centers such as point defects, grain boundaries along the growth direction detailed above, and possible interfacial rocksalt phase separation. Additional research will be needed to understand and overcome this effect. Nevertheless, unambiguous ferroelectric switching of Zn_1–*x*_Mg_*x*_O is demonstrated in films containing Mg concentrations of *x* = 0.46 and *x* = 0.58 prepared by ALD and shows that this conformal coating method can be used to prepare wurtzite ferroelectrics under BEOL-compatible conditions.

In summary, these results show that ALD can be used to incorporate large magnesium concentrations into Zn_1–*x*_Mg_*x*_O thin films and enable ferroelectric switching. The crystal structure becomes increasingly distorted and the bandgaps larger with greater magnesium content. Polarization reversal is demonstrated in the two highest magnesium content films (*x* = 0.46 and 0.58). This finding presents opportunities to further mature the Zn_1–*x*_Mg_*x*_O ALD processing space in which ferroelectric switching is possible, as well as explore ALD of other ferroelectric wurtzite families. Ultimately, the results herein represent a significant step toward the implementation of ferroelectric wurtzites into nonvolatile memory devices with high conformality requirements.

## Supplementary Material



## Notes

Additional information and data on material synthesis, characterization methods, X-ray diffraction patterns, lattice parameters, optical data, transmission electron microscopy, AFM, PFM, SEM, piezoresponse force microscopy, and microstructural data (PDF)
