# Supplementary material for: Ferroelectricity in Atomic Layer Deposited Wurtzite Zinc Magnesium Oxide Zn1–x Mg x O
Source: Nano Lett. 2025 Jun 9;25(24):9748–54. doi: 10.1021/acs.nanolett.5c02005 (PMC12186618; doi:10.1021/acs.nanolett.5c02005)
Supplement: Supplementary file 1 [file nl5c02005_si_001.pdf]

Ferroelectricity in Atomic Layer Deposited Wurtzite Zinc Magnesium Oxide $Zn_{1-x}Mg_xO$

Benjamin L. Aronson [a], *Kyle P. Kelley* [b], *Ece Gunay* [c], *Ian Mercer* [d], *Bogdan Dryzhakov* [b], *Jon-Paul Maria* [d], *Elizabeth C. Dickey* [c], *Susan Trolier-McKinstry* [d], *and Jon F. Ihlefeld* [a,e*]

[a] *Department of Materials Science and Engineering, University of Virginia, Charlottesville, Virginia 22904, United States*

[b] *Center for Nanophase Materials Sciences, Oak Ridge National Laboratory, Oak Ridge, Tennessee 37381, United States*

[c] *Department of Materials Science and Engineering, Carnegie Mellon University, Pittsburgh, Pennsylvania 15213, United States*

[d] *Department of Materials Science and Engineering, The Pennsylvania State University, University Park, Pennsylvania 16802, United States*

[e] *Charles L. Brown Department of Electrical and Computer Engineering, University of Virginia, Charlottesville, Virginia 22904, United States*

* Email: jfi4n@virginia.edu

Supporting Information

Synthesis of $Zn_{1-x}Mg_xO$ films

$Zn_{1-x}Mg_xO$ films were deposited onto 50 nm (111)-oriented Pt/5 nm Ti/SiO_2/(001)-oriented silicon substrates using an Arradiance GEMStar XT-P atomic layer deposition tool. Prior to deposition, the instrument was evacuated to a chamber pressure of 260 mTorr. A chamber and platen temperature of 175 °C was used for the growth, with the metal precursor manifold set to 125 °C and the secondary manifold maintained at 140 °C. Diethylzinc (DEZ) was used as the zinc precursor with a purity of 95% and bis(ethylcyclopentadienyl)magnesium ($Mg(CpEt)_2$) was used as the magnesium precursor with 98% purity. The DEZ and $Mg(CpEt)_2$ precursor cylinders were held at room temperature and 90 °C, respectively. The system was held at the selected deposition temperature for 20 minutes prior to the first dose to ensure a uniform deposition temperature. A continuous flow of 7 sccm of argon transported the metal precursors through the manifolds into the process chamber during the dose steps and removed reaction byproducts during the purge steps. An additional flow of 70 sccm of argon through the inductively coupled plasma head was used during the metal precursor dose and purge steps to minimize deposition into the plasma head. An argon:oxygen plasma (5 sccm Ar and 5 sccm O_2) was used as a coreactant to oxidize the metal precursor surface species. Prior to the plasma dose, the gas flows through the plasma were reduced from 70 sccm to the aforementioned flowrates with a 10 second delay to allow the flow and chamber pressure to stabilize. The forward and reflected powers during the plasma dose were 300 W and 5 W, respectively. The growth sequence of the ZnO and MgO layers consisted of 5 steps each: precursor dose, precursor purge, plasma stabilization, plasma dose, and plasma purge. The ZnO sequence timing was 0.03/24/10/10/5 s and the MgO sequence timing was 0.5/28/10/10/5

s. The supercycle ratio of ZnO:MgO was altered to achieve films of varying composition, with the compositions shown in **Figure S1**. Following the growth, the chamber and platen were cooled to a temperature of 75 °C before sample removal from the ALD instrument.

Characterization methods

X-ray diffraction (XRD) measurements were performed using a Rigaku Smartlab diffractometer with Cu-Kα radiation with a HyPix 3000-HE detector. All scans utilized a 5 mm mask and incident and receiving 5.0° Soller slits. Symmetric 2θ-ω scans were collected using a Bragg Brentano selection slit, 0.5° divergence slit, copper Kβ filter, step size of 0.02° and scan rate of 2°/minute. Rocking curve scans of the 0002 wurtzite $Zn_{1-x}Mg_xO$ and 111 platinum reflections were collected using a parallel beam selection slit, 1.0 mm divergence slit, step size of 0.04° and scan rate of 1°/minute. In-plane $2\theta_\chi$ scans of the $10\bar{1}0$ wurtzite $Zn_{1-x}Mg_xO$ reflection were collected using a parallel beam selection slit, 0.1 mm divergence slit, 0.5° parallel slit collimator, 0.5° parallel slit analyzer, incidence angle of 0.7°, step size of 0.008°, and scan rate of 0.5°/minute. Variable Angle Spectroscopic Ellipsometry was conducted using a Woolam M-2000 Ellipsometer. Scans of each sample were collected at angles of 55°, 60°, 65°, 70°, and 75°. Data was collected in the wavelength range of 196 nm to 1690 nm for each angle mentioned prior.

Cross-sectional TEM samples were prepared by wedge polishing, followed by Ar+ ion milling using a Gatan Precision Ion Polishing System (PIPS II). High-angle annular dark-field scanning transmission electron microscopy (HAADF-STEM) imaging was performed on an aberration-corrected Thermo Scientific Themis microscope, with a collection angle ranging from 46 to 200 mrad. Imaging was conducted at 200 kV, with a beam convergence angle of 25 mrad. Energy

dispersive X-ray spectroscopy (EDS) data was collected using a Super XG2 detector with a probe current of approximately 130 pA.

All piezoresponse force microscopy and spectroscopy measurements were acquired using single crystal diamond Adama probes (AD-2.8-AS) with an Oxford Instruments Vero quadrature phase differential interferometric AFM. All measurements were acquired in single frequency mode, off resonance at 72 kHz using a driving voltage of 1 V_{ac}. Switching spectroscopy was acquired using a stepped on and off triangle wave, as previously reported,[1] on the bare surface of $Zn_{1-x}Mg_xO$. For PFM poling, a DC power supply was connected to the AFM cantilever to supply ±16 V.

A TESCAN MIRA field emission Scanning Electron Microscope (FESEM) was used to image the films. A secondary electron (SE) detector with a 4 keV beam energy and 4 mm working distance was used to analyze surface morphology along with a backscatter electron (BSE) detector at 5 keV and a working distance of 8 mm to analyze phase contrast. To minimize surface charging, the sample was contacted with carbon tape, copper tape, and coated with 3 nm of tungsten. Prior to imaging, a 20 W oxygen plasma clean was performed in the SEM chamber for 5 minutes to minimize hydrocarbons on the sample surface.

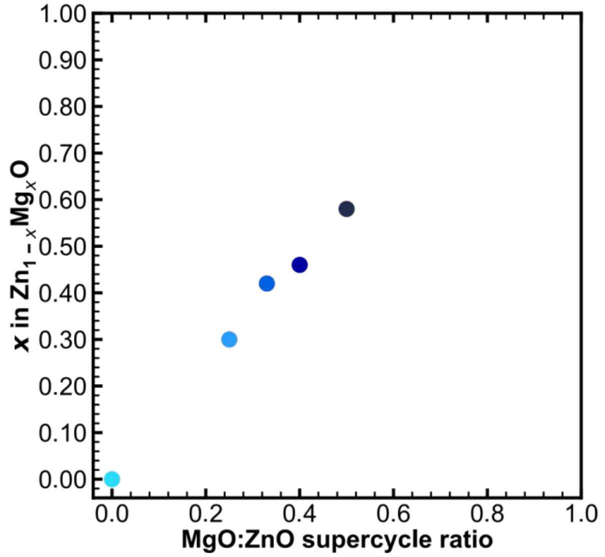

Figure S1. Magnesium content (x) derived from RBS measurements in $Zn_{1-x}Mg_xO$ films across the composition series as a function of the MgO:ZnO supercycle ratio utilized in the PE-ALD growth process. The error bars for composition are smaller than the size of the symbol.

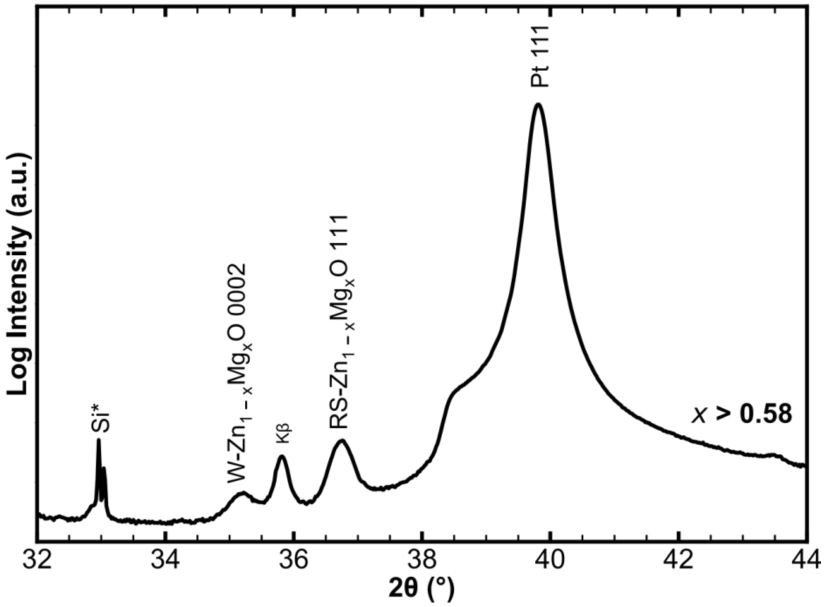

Figure S2. X-ray diffraction pattern of $Zn_{1-x}Mg_xO$ film with an average magnesium concentration greater than 58% ($x > 0.58$). The MgO:ZnO supercycle dose ratio used for the film growth was 2:1. Phase separation is evident due to the presence of both the wurtzite 0002 and rocksalt 111 reflections.

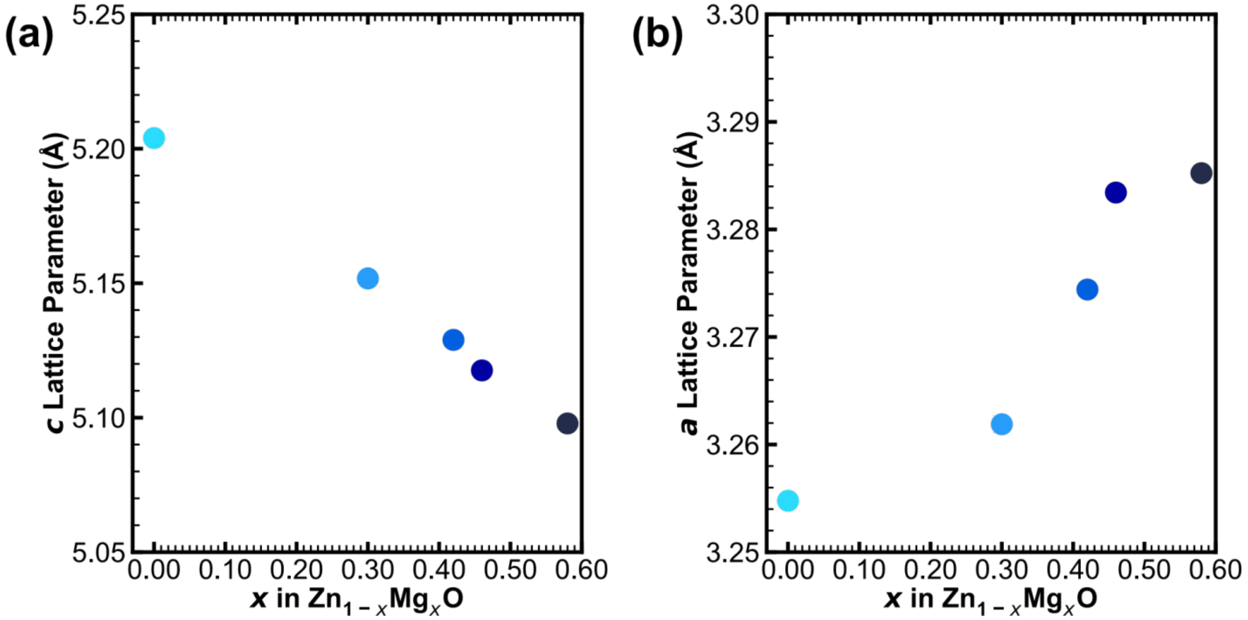

Figure S3. (a) *c* and (b) *a* lattice parameters of $Zn_{1-x}Mg_xO$ thin films across the composition series for $x = 0, 0.30, 0.42, 0.46, 0.58$.

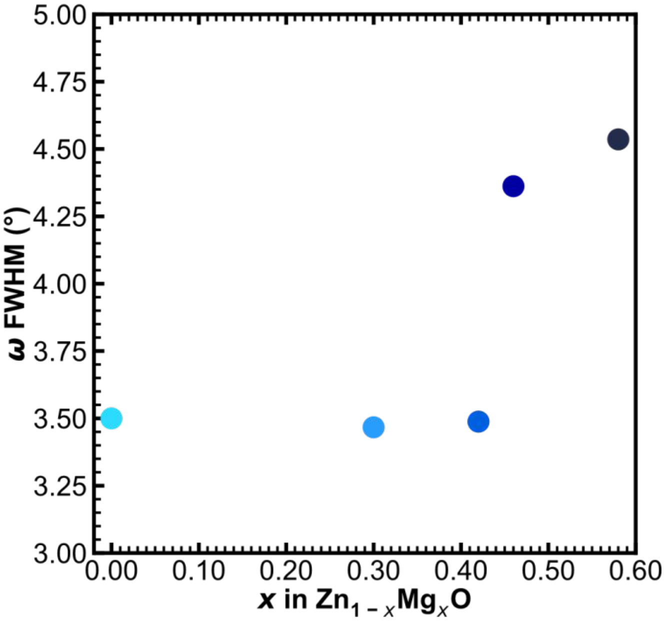

Figure S4. ω FWHM of $Zn_{1-x}Mg_xO$ thin films across the composition series for x = 0, 0.30, 0.42, 0.46, 0.58.

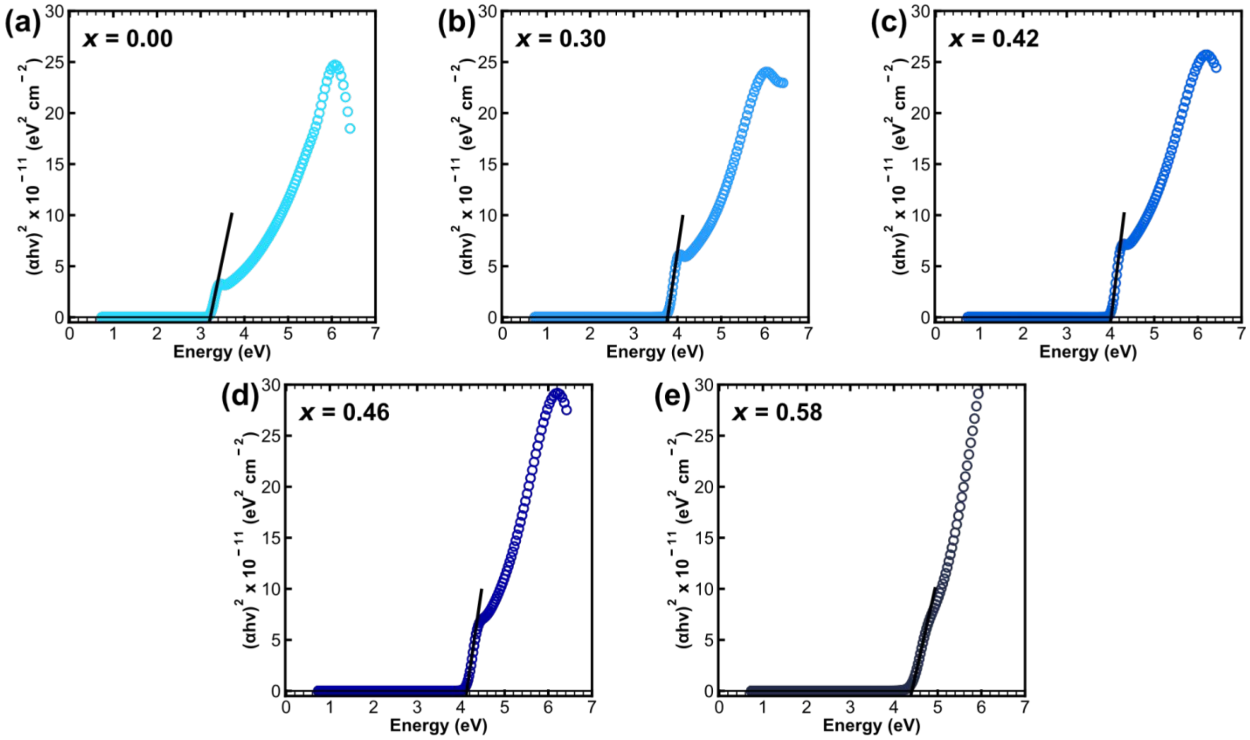

Figure S5. Tauc plots of $(\alpha h\upsilon)^2$ as a function of incident photon energy for $Zn_{1-x}Mg_xO$ films with magnesium concentrations of (a) x = 0, (b) x = 0.30, (c) x = 0.42, (d) x = 0.46, and (e) x = 0.58. The extrapolated x-intercept of the linear regression at the absorption edge is the direct optical bandgap energy.

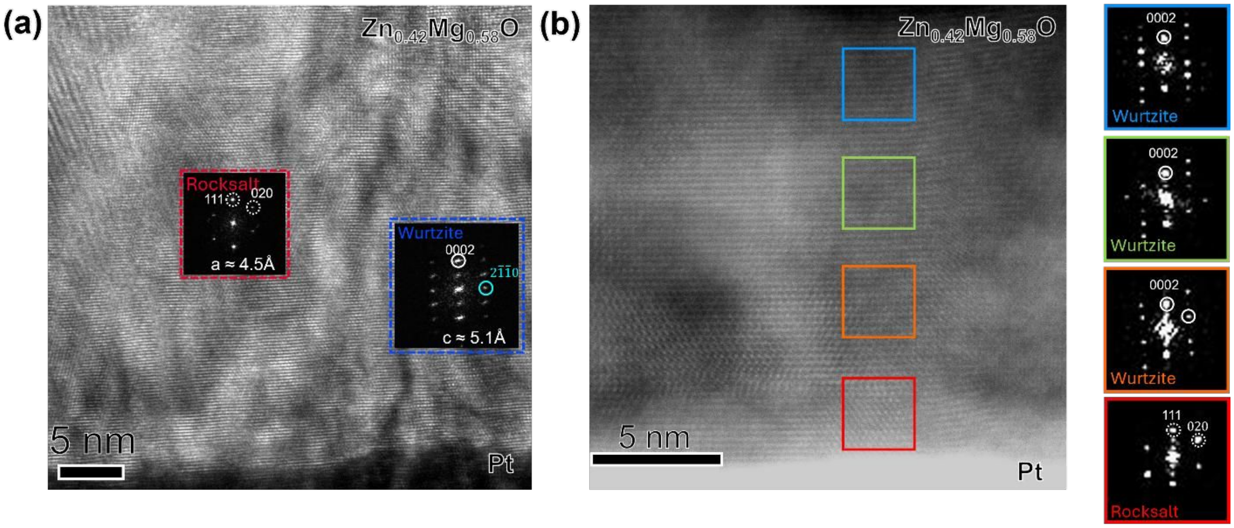

Figure S6. Local FFT images from different regions of the sample, confirming the presence of a secondary rocksalt phase. (a) HRTEM image showing a grain with cubic symmetry (red inset). (b) STEM image and local FFTs highlighting the presence of the rocksalt phase, despite wurtzite being the predominant structure.

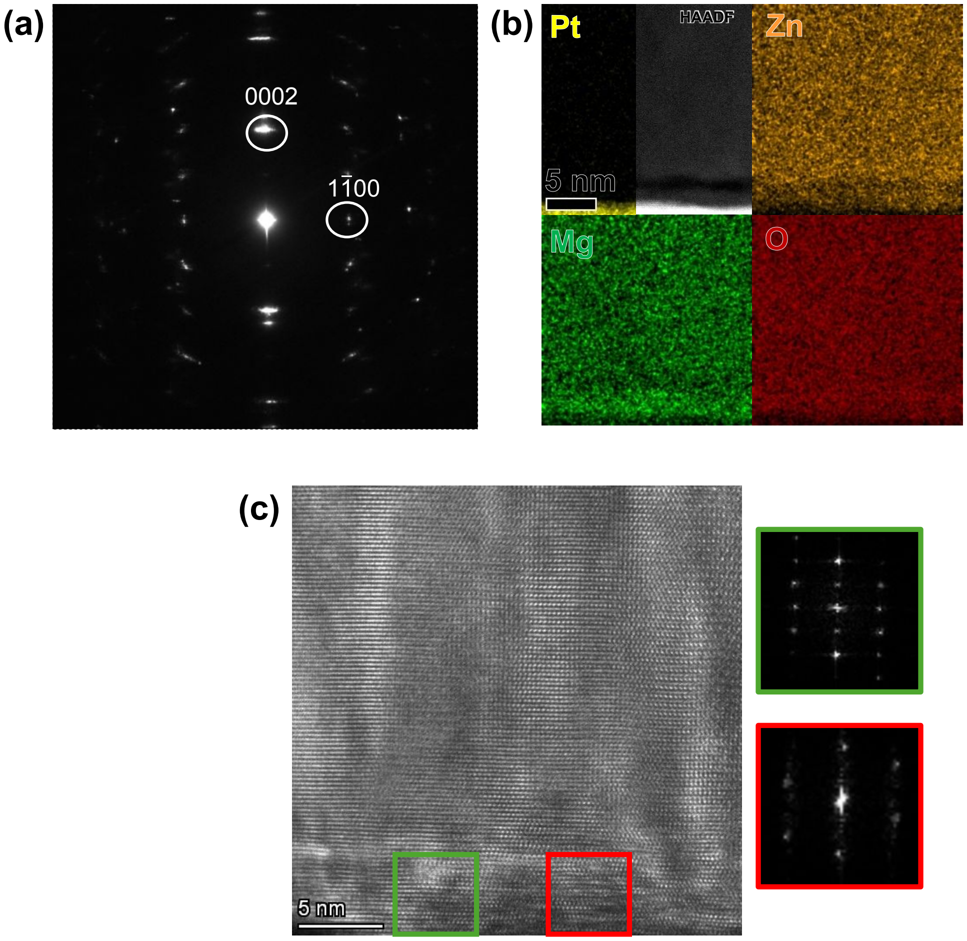

Figure S7. (a) Selected area diffraction pattern of the $Zn_{0.54}Mg_{0.46}O$/Pt/Ti/SiO_2/Si stack. (b) STEM-EDS elemental map indicating zinc and magnesium distribution throughout the thickness of the film. (c) HRTEM image and local FFTs highlighting the presence of the wurtzite (green) and rocksalt (red) phases near the interface between the $Zn_{0.54}Mg_{0.46}O$ and Pt substrate.

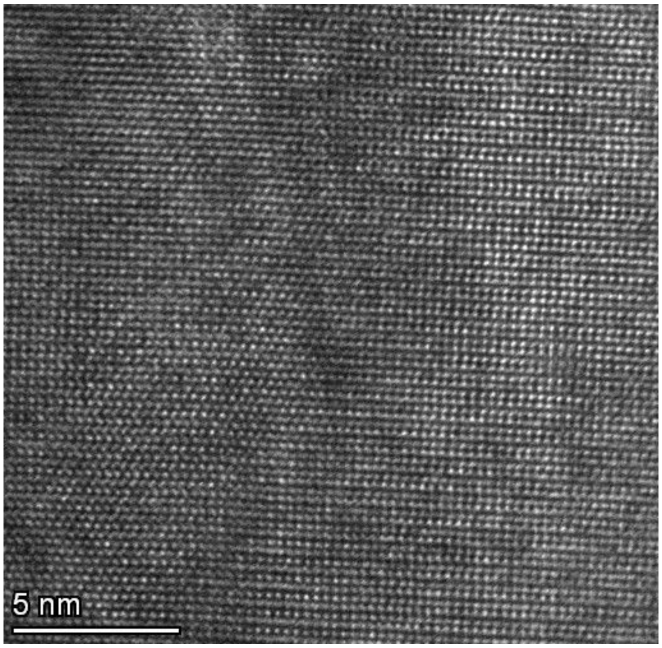

Figure S8. High resolution transmission electron microscopy (HRTEM) image of the $Zn_{0.54}Mg_{0.46}O$ film in the bulk region.

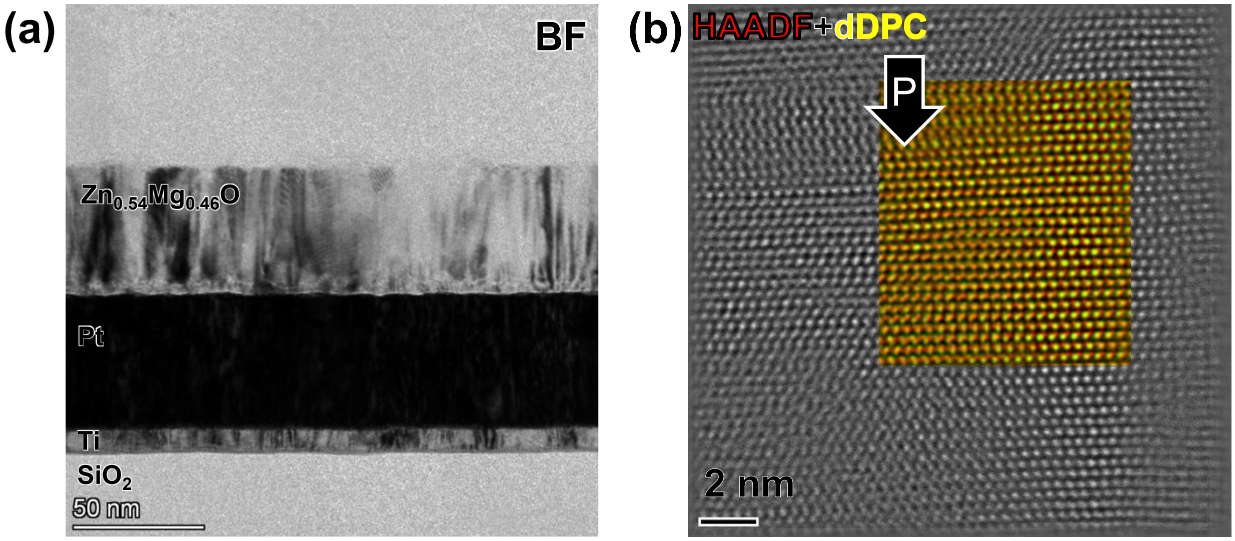

Figure S9. (a) Brightfield TEM image of the $Zn_{0.54}Mg_{0.46}O/Pt/Ti/SiO_2/Si$ stack. (b) STEM-HAADF atomic resolution image of the film structure, overlaid with dDPC(inset) showing the polar alignment of oxygen anions (red) and cations (yellow).

Table S1. Thickness, mean squared error, bandgap, refractive index, and extinction coefficients as a function of $Zn_{1-x}Mg_xO$ compositions as determined by VASE.

Mg concentration (x)	Thickness (nm)	Mean Squared Error	Bandgap (eV)	Refractive index (633 nm)	Extinction coefficient (633 nm)
0.00	55.1	4.96	3.2	1.96	0.006410
0.30	52.9	4.26	3.8	1.87	0.007417
0.42	51.2	4.50	4.0	1.83	0.004259
0.46	51.9	4.36	4.1	1.81	0.000397
0.58	53.5	5.60	4.4	1.80	0.000618

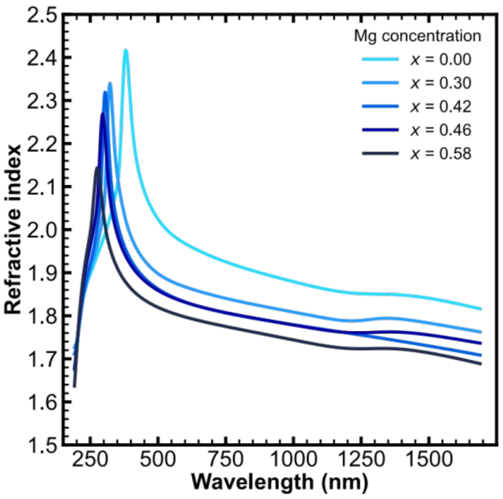

Figure S10. Refractive index versus wavelength across the composition series.

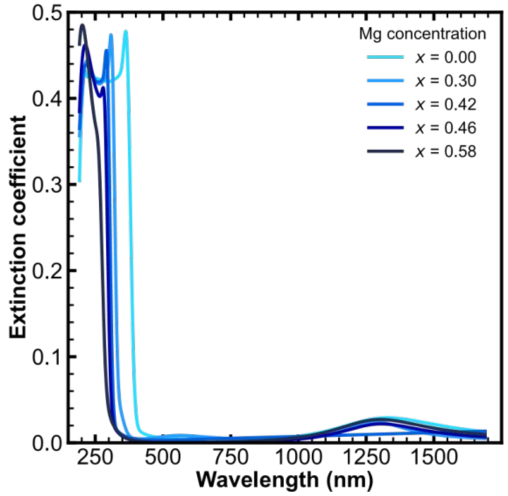

Figure S11. Extinction coefficient versus wavelength across the composition series.

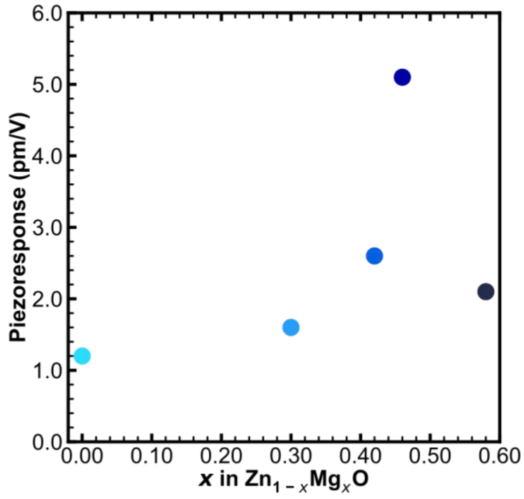

Figure S12. Average piezoresponse versus magnesium concentration across the composition series.

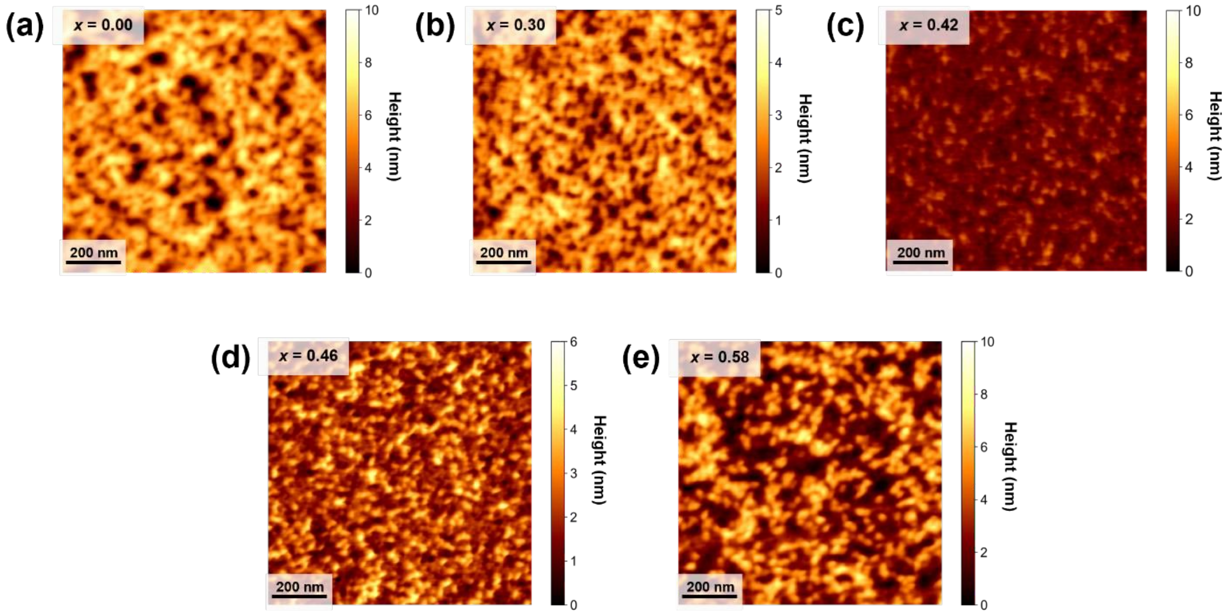

Figure S13. AFM topography images (1 μm^2) across the composition series.

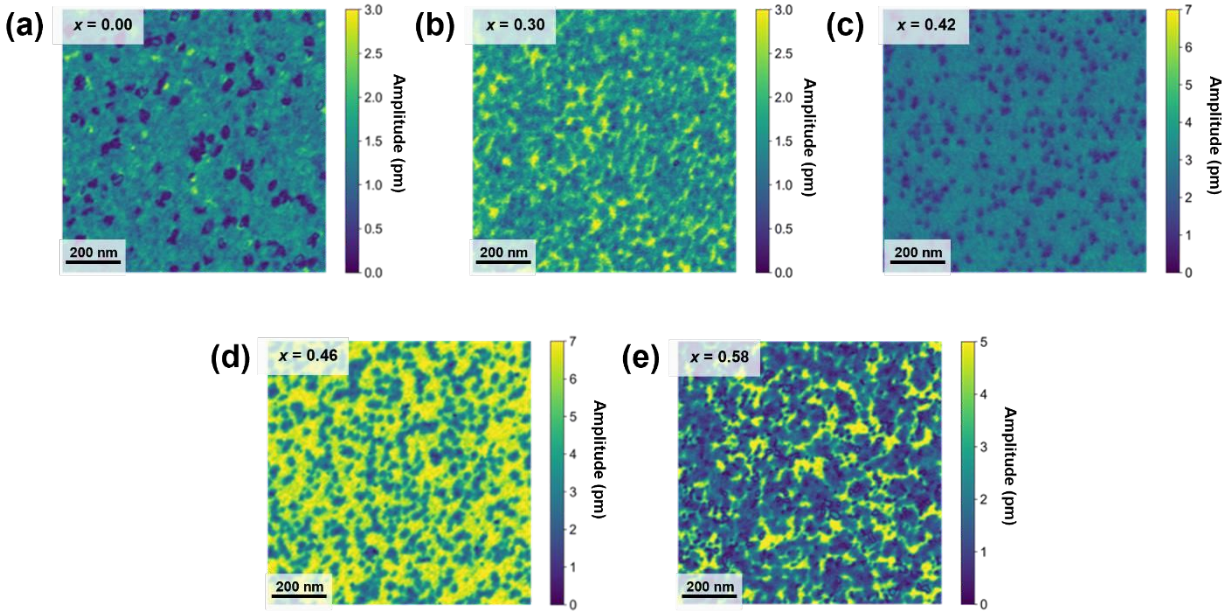

Figure S14. PFM amplitude maps (1 μm^2) across the composition series.

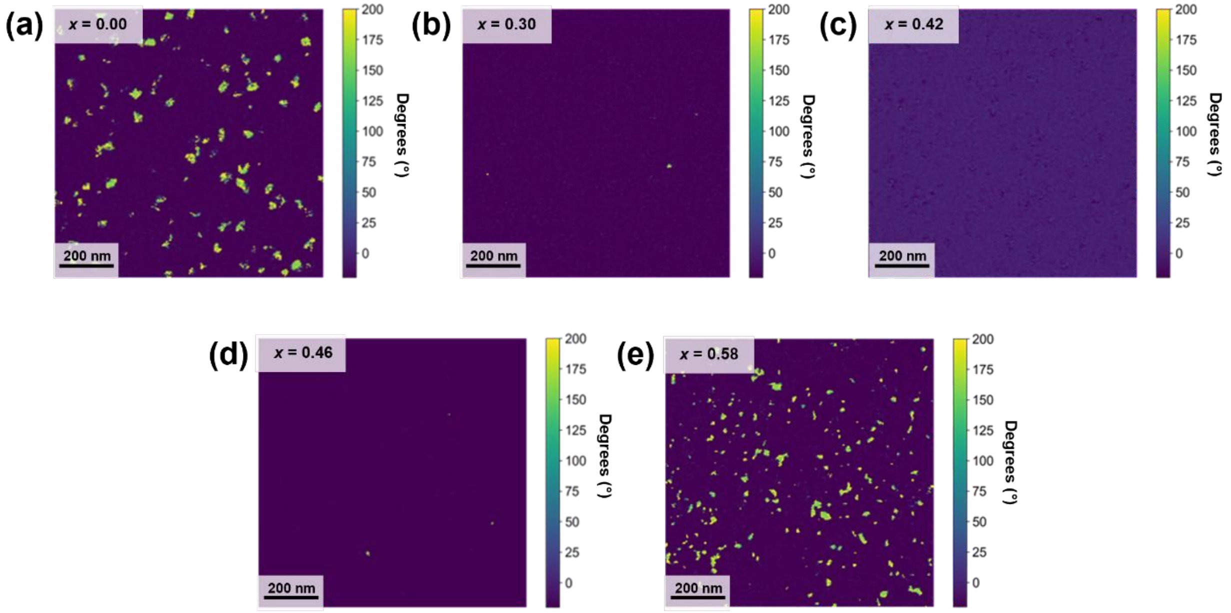

Figure S15. PFM phase maps (1 μm^2) across the composition series.

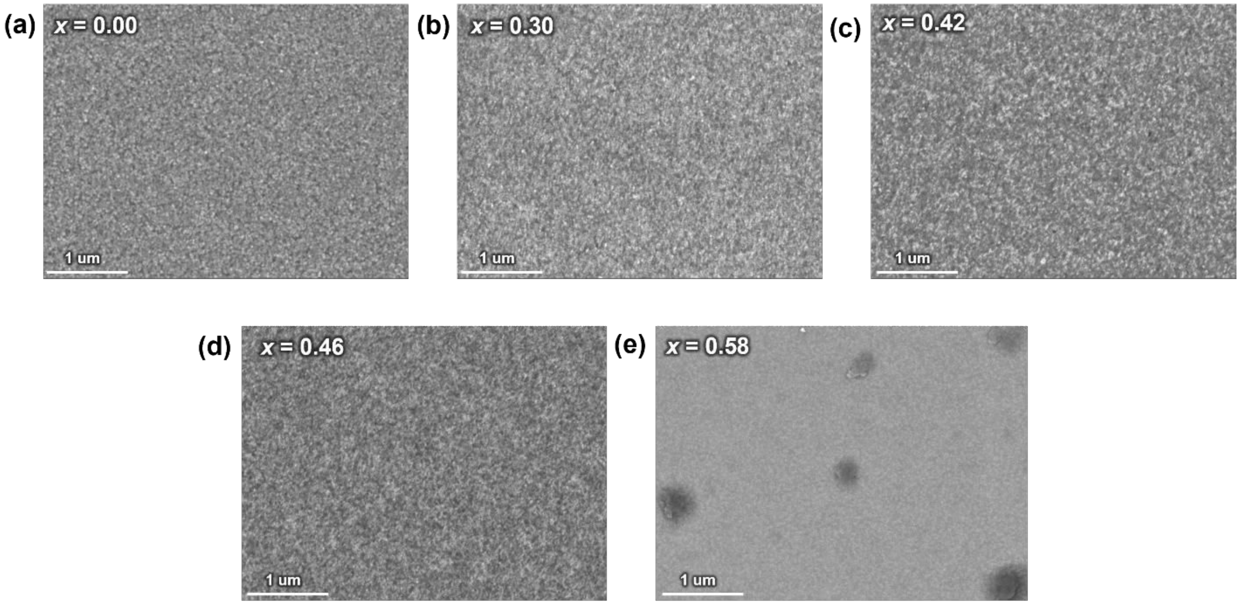

Figure S16. Backscattered electron SEM images of film surface across the composition series. Z-contrast consistent with composition segregation is observed in the highest magnesium containing film, $x = 0.58$.

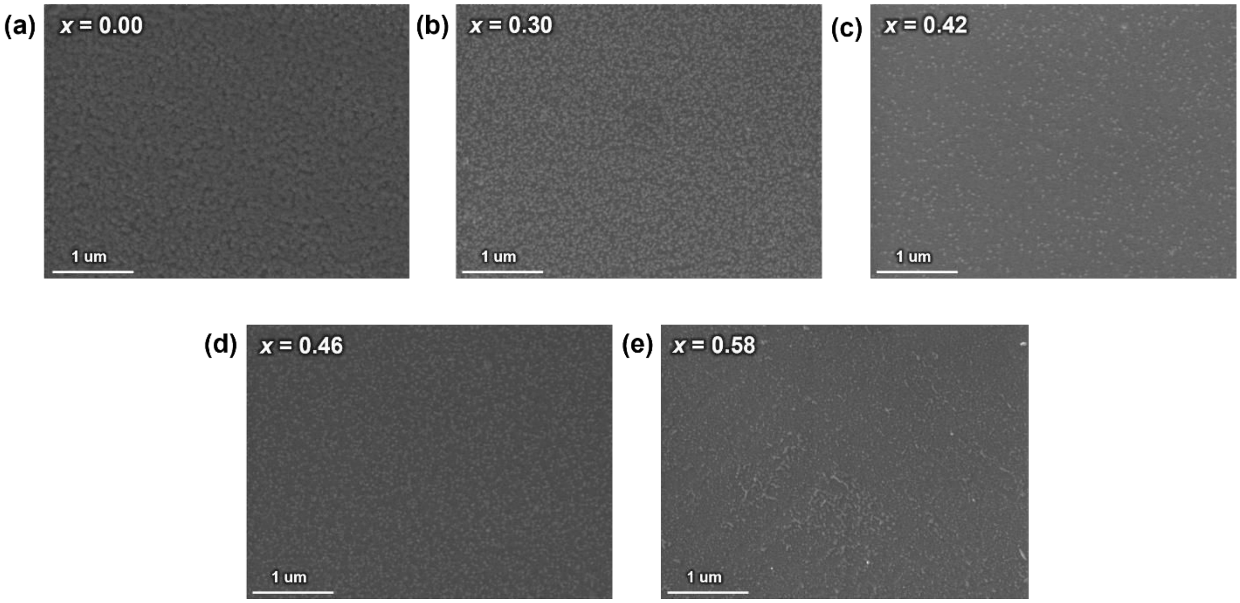

Figure S17. Secondary electron SEM images of film surface across the composition series.

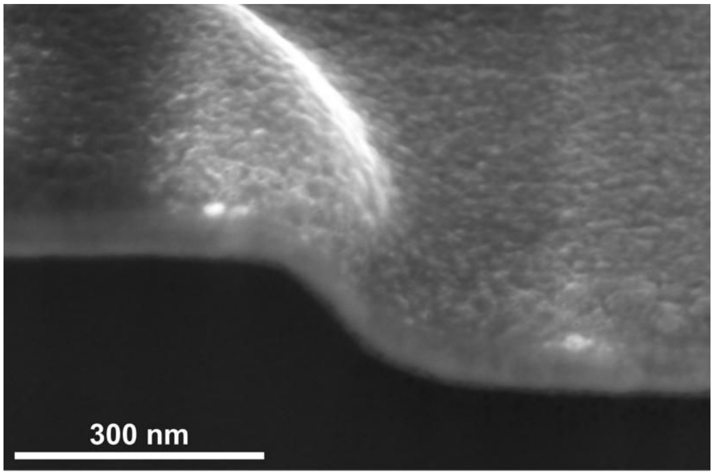

Figure S18. Cross-sectional secondary electron SEM image of a $Zn_{0.54}Mg_{0.46}O$ film deposited conformally on 3D platinized silicon pillars.

Author Contributions

The manuscript was written through contributions of all authors. All authors have given approval to the final version of the manuscript.

ACKNOWLEDGMENT

This material is based upon work supported by the Center for 3D Ferroelectric Microelectronics Manufacturing (3DFeM2), an Energy Frontier Research Center funded by the U.S. Department of Energy, Office of Science, Office of Basic Energy Sciences Energy Frontier Research Centers program under Award Number DE-SC0021118. The authors acknowledge Applied Materials, Inc. for providing the platinized silicon used in this study. RBS measurements were performed by Daniele Cherniak at the University of Albany, State University of New York. The piezoresponse force microscopy research was supported by the Center for Nanophase Materials Sciences (CNMS), which is a U.S. Department of Energy, Office of Science User Facility at Oak Ridge National Laboratory.

References

(1) Jesse, S.; Baddorf, A. P.; Kalinin, S. V. Switching Spectroscopy Piezoresponse Force Microscopy of Ferroelectric Materials. *Appl. Phys. Lett.* **2006**, *88* (6), 062908. DOI: 10.1063/1.2172216.
